# Surgery

**Published:** 1900-06

**Authors:** C. A. Morton


### SURGERY.

In writing the "progress of Surgery" for this number of the *Journal*, it seems to me that it would be well to give a short account of the more recently published results of the surgery of **malignant disease in the alimentary canal.** When a patient asks our advice as to the removal of an innocent tumour, only three questions need consideration—whether it need be removed, whether the whole growth can be removed without too serious a risk, and the fitness of the patient for any operation. But when we have to advise as to the removal of a malignant tumour we have also to consider the chance of a recurrence after removal, and the probable rapidity of its onset; and if it recurs, whether the recurrence is likely to be as painful or as quickly fatal as the primary growth. In balancing the patient's chances in our mind, we have to throw the risks of recurrence into the scale along with the risks of operation, and only when the chances of prolongation of life or cure of the disease weigh them down shall we be able to recommend operation. That we must be able to get well beyond the growth in its removal is an essential condition of success, for the tissue surrounding it is almost certainly invaded by growth which the microscope would reveal.

I shall endeavour to answer the following questions with regard to the forms of malignant growth considered: 1. What is the outlook for the patient without operation? 2. What is the risk of removal of the growth? 3. What chance is there of freedom of recurrence? 4. If the disease recurs after operation, is it likely to be less painful or less rapidly fatal than the primary growth?

The prospects after operation for cancer of the tongue were considered in my Surgery Periscope for last June.

**Carcinoma of the stomach.**—The duration of life after a diagnosis of cancer of the stomach can be made is not long. In 26 cases of Krönlein's, seen before the disease was very advanced, the average duration of life was about eight months.[1] Hale White[2] says "the duration of the greater number of cases is much less than eighteen months, and often is less than a year." During the period that the patient survives his sufferings are often great. As a rule the growth is situated at the pylorus, and can be removed by resection of this portion of the organ. The operation is now by no means the desperate measure it was ten years ago, indeed the latest statistics of the operation are distinctly encouraging. In 1898 Krönlein published[3] a consecutive series of 24 cases of pylorectomy with five deaths, and only two deaths in the last 20 cases. He selected his 24 cases very carefully out of 67 cases seen by him. In 22 cases he explored only and did not remove the growth. Kocher has published his last series of 23 cases of pylorectomy with only two deaths.[4] Kocher states that carcinoma of the stomach is now a curable disease, and patients from whom it has been removed may live for years, well nourished and with a good digestion.[5] He has had a case of survival for nine years after pylorectomy, and there are several cases of freedom from recurrence for three years after operation.

If when the abdomen is opened pylorectomy is not found to

[1] Quoted by Barker, *Brit. M. J.*, 1899, i. 1267.

[2] Clifford Allbutt's *System of Medicine*, vol. iii., 1897, p. 566.

[3] Quoted by Barker, *Brit. M. J.*, 1899, i., 1266.

[4] *Ibid.* [5] *Brit. M J.*, 1898, ii. Epitome, p. 105.

be possible, on account of adhesions or secondary growths in the liver or glands, an opening can be made between the stomach and jejunum (gastro-enterostomy) in order to relieve the gastric symptoms, especially in cases of considerable pyloric obstruction. Life is not, however, likely to be nearly as much prolonged by this operation as by pylorectomy. Thus Stendel (from Czerny's clinic) gives the mortality of the operation for cancer of the stomach as 34.5 per cent., and for other diseases as 14.3 per cent.[1] Stendel also gives the average duration of life after gastro-enterostomy for cancer as 12.6 months but if cases of doubtful diagnosis, in which life has been prolonged for years, are excluded, as seven to eight months.[2]

There are eight cases of complete removal of the stomach for malignant disease with a mortality of 50 per cent., and 14 cases in which three-quarters of the stomach have been removed with a mortality of 28.5 per cent.[3] In my own case of resection of three-quarters of the stomach for sarcoma, the patient reported himself as quite well in February of this year, *i.e.* eight months after operation.

**Carcinoma of the colon.**—Maylard[4] says: "It is usual for death to occur in uncomplicated cases in less than a year from the onset of the earliest symptoms." But very often the earliest symptoms of growth in the colon are those of intestinal obstruction. Resection of a growth in the colon used to be a very dangerous operation, and still involves considerable risk. Kendal Franks in 1889 communicated to the Royal Medico-Chirurgical Society statistics of a large number of these cases with a mortality of 40 per cent.[5] Some much more encouraging statistics have of late years been published[6] by the surgeons of the Leeds Infirmary. In 26 cases the mortality was about 28 per cent. In nine cases of simple suturing five died; in five cases in which the Murphy button was used only one died; and out of 12 cases in which the decalcified bone bobbin was used only one died.

There are cases on record of non-recurrence for a considerable length of time. The patient from whom I excised the whole cæcum and some glands for carcinoma in November, 1898, is now in perfect health.

**Cancer of the rectum.**—The average duration of life in cancer of the rectum after the first symptom has appeared is two years, and, like cancer of the tongue, the disease causes great distress in its later stage. This distress can generally be relieved by colotomy, which when performed for this purpose has a mortality of only 2 or 3 per cent. Obstruction may be relieved by colotomy also, but in this condition there is a higher mortality. Pain from infiltration of the base of the bladder or sacral nerves

[1] Quoted by Mayo Robson, *Lancet*, 1900, i. 833. [2] *Ibid.*
[3] *Ibid.*, 842.
[4] *The Surgery of the Alimentary Canal*, 1896, p. 471.
[5] *Med.-Chir. Tr.*, 1889, lxxii. 211. [6] *Brit. M. J.*, 1896, i. 845.

would not be relieved by colotomy. The mortality of excision of the growth varies greatly in the practice of different surgeons, and has as a rule been greater if the disease has been situated high up in the rectum and can only be removed by the sacral route, with perhaps a free opening in the peritoneum (Kraske's operation). The old limitation of operation to cases in which the growth was confined to the lower end of the bowel and could be removed without opening the peritoneum has now been abandoned by many surgeons,[1] and though as a rule Kraske's operation for removal of cancer situated high up has a higher mortality than the perineal operation, this has not been the case in the hands of some surgeons. Although in Kraske's first 29 cases the mortality was as high as 34.5 per cent., after improving his method, in his last 51 cases it was only 9.8 per cent.[2] Swinford Edwards has operated on 14 cases by Kraske's method with only 14 per cent. mortality.[3] Ball has operated on 17 cases by the sacral method (Kraske's) with only one death.[4] but as he is in favour of this method rather than the perineal, and would not therefore reserve it for cases of cancer high up in the bowel, probably several of his cases did not necessitate as severe an operation as is usually the case where Kraske's method is adopted. Recognising this however, a mortality of 5 or 6 per cent. is very good, even for perineal operations.

The results of excision of rectal cancer eight years ago were worked out in a very exhaustive paper by McCosh of New York in 1892.[5] He collected statistics of 375 operations, some of which were by the perineal route and some by Kraske's method. They were nearly all from the records of German surgeons. The mortality was 19.1 per cent., and there were 11 per cent. of cures. At the present time the mortality of the perineal operation in cases in which the disease has not invaded the neighbouring parts is about 15 per cent.[6] In any series of cases of Kraske's operation it has seldom been more than 25 per cent., and, as already mentioned, in Kraske's last 51 is as low as any favourable cases would give by the perineal route. Paul[7] has excised cancerous growths from the bowel in 24 cases, and in 16 of these by Kraske's method, with a mortality of 16 per cent. Three of the deaths were from Kraske's operation.

If then we take the mortality after excision of the growth to

[1] Mr. Swinford Edwards (*Brit. M. J.*, 1897, i. 1210) says: "I know of three or four cases in my own practice submitted to excision and now well and engaged in their various occupations without any sign of recurrence after periods varying from one and a-half to six years, who have been advised by hospital surgeons of repute that the disease did not admit of removal, and that colotomy was the only thing left for them."

[2] *Ann. Surg.*, 1897, xxvi. 371. [3] *Loc. cit.* [4] *Brit. M. J.*, 1897, ii. 1069.

[5] *N. York M. J.*, 1892, lvi. 259.

[6] Ball, *The Rectum and Anus*, 2nd Ed., 1894; Treves's *System of Surgery*, 1895-96; and Platt, *Med. Chron.*, 1894, N.S. i. 419.

[7] *Lancet*, 1897, ii. 78.

be about 15 per cent., what advantage has the operation to offer compared with the risk? Colotomy, with almost no mortality, will probably relieve the patient's distress. Will excision of the growth give him any considerable chance of cure, or of prolongation of life for any considerable time? This question has been very fully discussed by Watson Cheyne.[1] He alludes to the hopeful fact that affection of glands and metastatic deposits are of late occurrence in cancer of the rectum,[2] unless the disease is unusually rapid in its course, and he points out that recurrences after operation frequently do not take place for several years, and quotes Czerny's statistics, which give 10 cases of non-recurrence for three years and over. Paul has also had three cures in 14 cases.[3] Watson Cheyne only recommends excision of the growth in the most favourable of cases; but we must take into consideration the fact mentioned by him that in Germany the radical operation is carried to an extreme extent, and adhesions to the bladder and prostate are not considered contra-indications, and Genzmer goes so far as to advocate the operation although metastatic deposits in the liver are present. This would give us a much smaller mortality for properly selected cases, for I think most of us would agree with Watson Cheyne that infiltration of neighbouring parts such as the bladder and prostate would be a distinct contra-indication to operation. Keen[4] has had 33 per cent. of cures in 15 cases of excision of cancer of the rectum, and he thinks patients live 18 months longer than they do after colotomy. Allingham,[5] who has performed excision of cancer of the rectum 62 times, draws attention to the fact that the older the patient the less the chance of recurrence after operation. He says that over 60 is "extremely favourable in suitable cases;" but if under 45 he thinks rapid recurrence certain, even if the growth does not extend much above the anus.

I have during the past six months operated on three cases of rectal carcinoma by the posterior or sacral method. In the first case the growth was very high up, and there had been some suppuration between it and the back of the uterus, which caused adhesions there. The peritoneum was freely opened, and after excision of the growth, the two ends of the rectum were sutured together. The posterior part of the line of sutures gave way and fæces discharged through this opening, but eventually it very greatly contracted, and the passage of a considerable quantity of fæces took place through the anus.[6] In the second case the growth was not quite so high, but was high enough to

[1] *Brit. M. J.*, 1896, i. 581.

[2] As to absence of metastatic growths, see also report (*Brit. M. J.*, 1900, i. 1031) of statement by Krönlein at the Surgical Congress, that in nearly 50 per cent. no metastasis was found *post mortem*.

[3] *Loc. cit.* [4] *Therap. Gaz.*, 1897, xxi. 225. [5] *Lancet*, 1896, i. 1133.

[6] I hear the patient has resumed her work as a charwoman, and that there is very little fæcal discharge from the sacral fistula.

require a free opening of the peritoneum. The divided rectum was sutured as in the first case, but also gave way posteriorly. No peritoneal or pelvic complication arose, but great restlessness which set in early after the operation (without high temperature), developed into insanity and he died exhausted some weeks later. In the third case the growth was not high enough to require an opening into the peritoneal cavity, or more than resection of the coccyx; but by means of suturing the divided rectum an almost perfect result has been obtained, the anus is normal, the posterior wound has soundly healed, and the only abnormal condition present is a need to hurry when he feels a desire to evacuate the bowels, due to a somewhat relaxed condition of the sphincter ani.

C. A. Morton.

